# Osteomyelitis and pyoarthritis resulting from local paracoccidioidomycosis in an immunocompetent patient: a case report

**DOI:** 10.1186/1752-1947-6-342

**Published:** 2012-10-08

**Authors:** Michel Santana Michelan, Eloy de Ávila Fernandes, Leonardo Furtado Freitas, Rodrigo Hoeller Ribeiro, Marília Magri Milano, Soraya Silveira Monteiro

**Affiliations:** 1 Radiology, Hospital do Servidor Público Estadual (HSPE/IAMSPE), São Paulo, SP, Brasil; 2 Fundação Instituto de Diagnóstico por Imagem (FIDI) at the Hospital do Servidor Público Estadual (HSPE/IAMSPE), São Paulo, Brasil; 3 Rua Dr. Gabriel dos Santos, 179 apto 41, Bairro Santa Cecília, CEP 01231-011, São Paulo, SP, Brasil; 4 Pediatrics, Hospital do Servidor Público Estadual (HSPE/IAMSPE), São Paulo, SP, Brasil

**Keywords:** Osteomyelitis, Paracoccidioidomycosis, Children

## Abstract

**Introduction:**

Paracoccidioidomycosis is a type of mycosis that is endemic to Brazil and is triggered by the fungus *Paracoccidioides brasiliensis*. Isolated bone involvement in this disease is very rare, especially in children. To the best of our knowledge this report documents the first case of an immunocompetent pediatric patient in which paracoccidioidomycosis of the hip articulation was the sole manifestation of the disease (that is, there were no pulmonary or skin lesions).

**Case presentation:**

An 11-year-old Brazilian Caucasian boy from a rural area was examined in the orthopedic ward of our emergency department. Our patient reported a three-month history of pain in the right hip with intermittent claudication and also complained of recurring episodes of intense pain and an inability to walk, which he had been experiencing for the previous five days. He additionally presented with a fever that had persisted for two days. Our patient’s medical history did not include any clinical respiratory manifestations, skin lesions, history of trauma or immunosuppression risk factors.

**Conclusions:**

This is one of the very few reported cases of isolated articular involvement in osteomyelitis in a pediatric immunocompetent patient. Paracoccidioidomycosis should be considered among the differential diagnoses in such cases, especially in cases of patients who reside in rural areas where the condition is considered to be endemic, in order to administer the proper course of treatment in a timely fashion and improve the chances of a favorable prognosis.

## Introduction

Paracoccidioidomycosis is a type of mycosis caused by the fungus *Paracoccidioides brasiliensis*, and although it is more commonly found in the skin, mucous membranes and lungs, it can also affect any organ or tissue. Cases of the disease are evenly distributed within areas considered to have high and low degrees of endemicity, such as Brazil, primarily among adult men who reside in rural areas and whose clinical characteristics are quite varied. According to data from epidemiological surveys carried out in relation to paracoccidioidomycosis in Brazil, Venezuela, Colombia and Argentina, it is believed that around 50% of the inhabitants in areas where the disease is endemic have been exposed to the causative agent of this mycosis. Fortunately, very few people develop any clinical manifestations. The highest risk factors for contracting this infection are related to activities involving management of soil that is contaminated with the fungus, such as agricultural activities, grading, soil preparation, gardening practices and transportation of plant products, among others. In adults, the predominant clinical presentation is chronic whereas in children or adolescents it is usually found to be acute or subacute. Adolfo Lutz initially described the disease in 1908. Due to the fact that bone paracoccidioidomycosis can be overlooked or confused with other more common diseases, such as neoplasms or bacterial infections, the diagnosis of osteoarticular infections caused by the fungus can be somewhat difficult. Furthermore, isolated bone involvement is very rare, especially in children. This mycosis poses a serious threat to public health due to its highly debilitating potential and the resulting number of premature deaths. Without proper diagnosis or appropriate treatment it can lead to a variety of severe and deadly forms in which there is rapid and progressive involvement of the lungs, integument, lymph nodes, spleen, liver and lymphoid organs of the digestive tract.

For this reason we have reported this rare case of an immunocompetent pediatric patient in which paracoccidioidomycosis of the hip articulation was the only manifestation of the disease (that is, there were no pulmonary or skin lesions).

## Case presentation

An 11-year-old Brazilian Caucasian boy from a rural area was examined in the orthopedic ward of our emergency department. Our patient reported a three-month history of pain in the right hip with intermittent claudication, and complained of recurring episodes of intense pain and an inability to walk, which he had been experiencing for the previous five days. He also presented with a fever that had persisted for two days. Our patient’s medical history did not include any clinical respiratory manifestations, skin lesions, history of trauma or immunosuppression risk factors.

**Figure 1:**
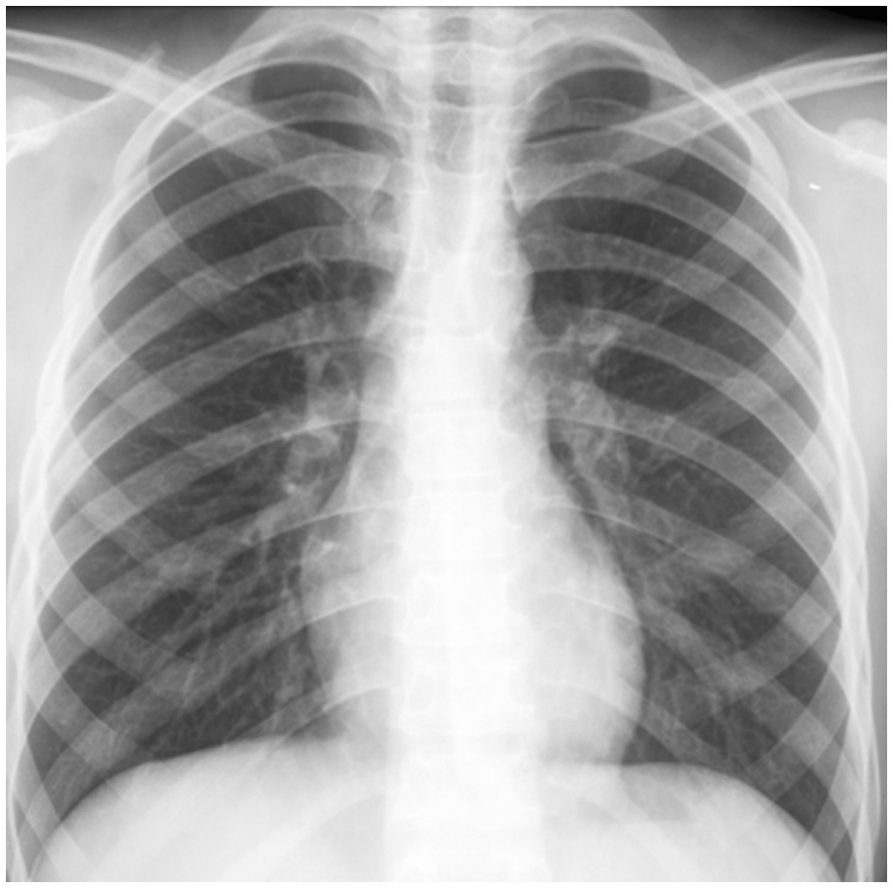
Chest radiograph without evidence of pulmonary involvement.

**Figure 2:**
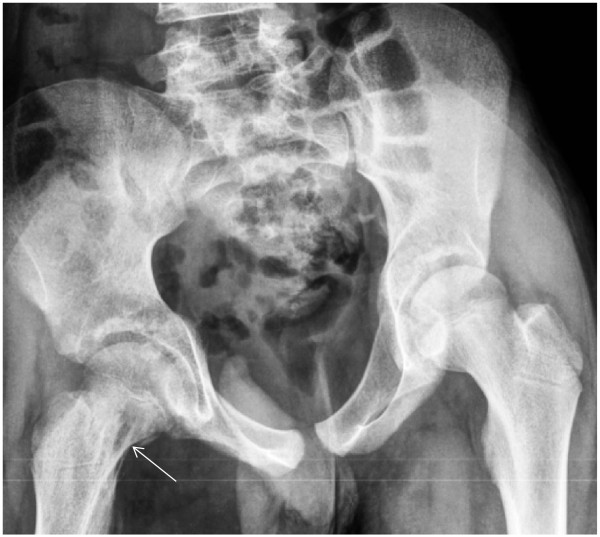
Radiograph of the pelvis showing an osteolytic lesion in the meta-diaphyseal region of the right femur with cortical discontinuity and increased volume and soft tissue density in the intra-articular projection (arrow), which is consistent with the existence of an effusion.

**Figure 3:**
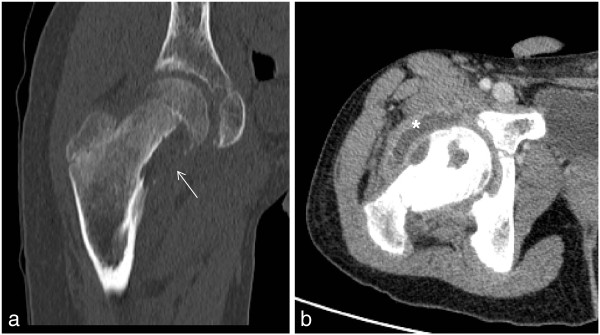
(A,B) Computed tomography scans of the right hip in the coronal (A) and transverse (B) planes demonstrating (A) an osteolytic lesion in the femoral neck (arrow), with partially enclosed margins and undefined areas of cortical bone, and (B) hypodense intra-articular material consistent with an effusion (*).

**Figure 4:**
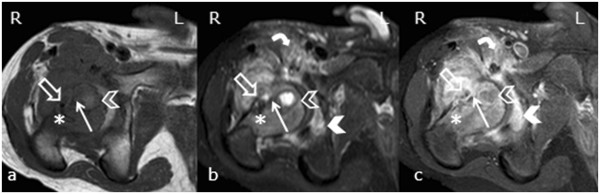
**Magnetic resonance imaging of the right hip with axial T1-weighted sequences (A), T2-weighted sequences using a fat suppression signal (B) and T1-weighted sequences after an intravenous injection of paramagnetic contrast medium (C), showing an area of T1 hypointensity and T2 hyperintensity in the meta-diaphyseal region of the femur with diffuse enhancement via the paramagnetic contrast medium (arrow) and a peri-osteal reaction on the medial face (open arrow).** Joint effusion and synovitis (closed arrowhead) are evident. The surgical path (*) can be observed in the lateral margin of the proximal femoral metaphysis for the biopsy site. Intra-osseous collection in the meta-diaphyseal region (open arrowhead) and edema of the iliopsoas muscle belly (curved arrow) are also visible.

A chest radiograph image (Figure 1) appeared normal, although a pelvis radiograph (Figure 2) showed an osteolytic lesion in the meta-diaphyseal region of the right femur and increased volume and soft tissue density in the intra-articular projection, which is consistent with the existence of an effusion. A computed tomography (CT) scan of the right hip showed an osteolytic lesion in the meta-diaphyseal region of the femur that was associated with a disruption of the cortical bone and hypodense intra-articular material that is consistent with an articular effusion (Figure 3). From a magnetic resonance imaging (MRI) study, osteomyelitis and pyoarthritis in the meta-diaphyseal region of the femur were characterized by an area of low signal intensity on the T1-weighted image. The presence of diffuse enhancement was visualized with the use of a paramagnetic contrast agent and the existence of a periosteal reaction was identified in addition to joint effusion with the enhancement, indicating synovitis and edema of the adjacent muscle bellies (Figure 4).

**Figure 5:**
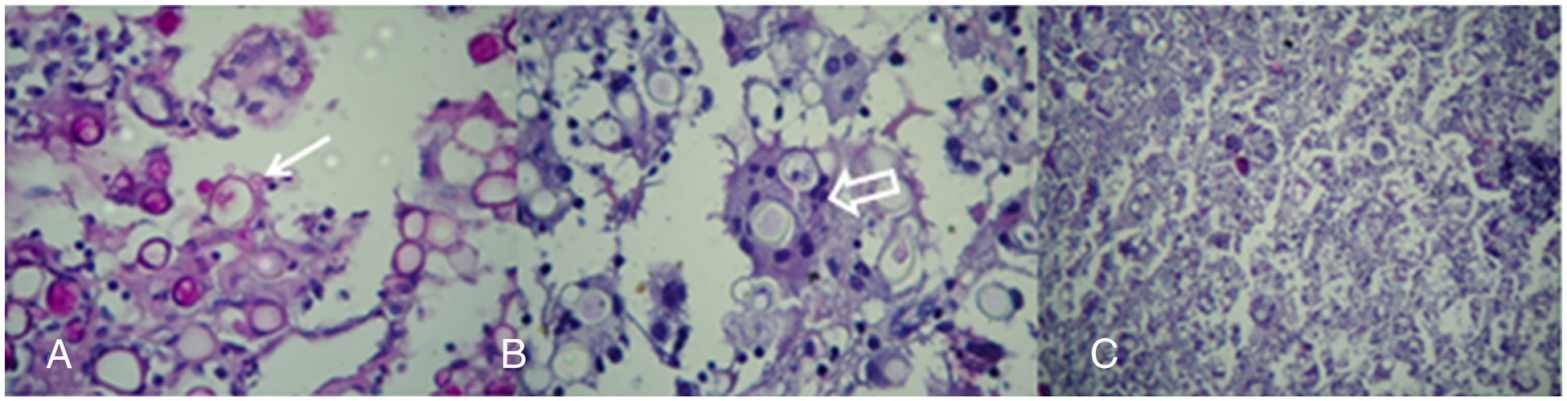
**(A) *Paracoccidioides brasiliensis *** **with multiple peripheral buds in the typical ‘pilot wheel’ configuration (arrow) (periodic acid-Schiff stain, 400×); (B) Birefringent rings, some of which show lateral budding cells being phagocytosed by multinucleated foreign-body giant cells (open arrow) (hematoxylin and eosin stain, 400×); and (C) the chronic granulomatous inflammatory process characterized by epithelioid cells and multinucleated giant cells surrounding the fungi (hematoxylin and eosin stain, 100×).**

Pyoarthritis drainage and a bone biopsy were performed and intravenous antibiotics were administered to our patient. The results of a pathological study confirmed the presence of *P. brasiliensis* (Figure 5) and fluconazole treatment was initiated (10mg/kg/day). Our patient is at the time of writing still undergoing treatment and showing significant improvements, confirmed by a reduction of laboratory markers of inflammatory/infection status: leukocyte count (10,000 to 6000; reference value <12,000); C-reactive protein (1.3 to 0.9mg/dL, reference value <0.6mg/dL), and erythrocyte sedimentation rate (120 to 60mm/hour; reference value <15mm/hour, for age and sex). He was discharged from hospital and returned one week later for reassessment.

## Discussion

Paracoccidioidomycosis in adults typically affects men more frequently than women at a ratio of 25:1, whereas this ratio among children is 1:1. In addition, the disease is more common among adults and there have been few published reports of pediatric cases. In 1967, Lacaz recorded 2902 cases of paracoccidioidomycosis, of which only 1.5% involved children. Castro and del Negro found that, of the 1899 patients admitted between 1944 and 1974, only 3.68% were under the age of 14 years [1-3].

Most osteoarticular lesions caused by *P. brasiliensis* are observed as a disseminated form of the disease and involve multiple organs. In contrast, isolated forms of *P. brasiliensis* osteoarticular involvement are rare and seldom reported in the literature. In a meta-analysis of studies selected from the Medline, Embase and Lilacs databases for the period between 1966 and 2001, Figueiredo *et al*. [4] examined 345 cases of fungal joint infections and found that only five of these cases resulted from *P. brasiliensis*. Amstalden *et al*. [5] published nine cases of paracoccidioidomycosis in which the musculoskeletal system was the primary location of disease manifestation and only two of these cases, one involving a femur and the other an elbow, showed no organ lesions.

A diagnosis of paracoccidioidomycosis is confirmed when suppurative granulomas with giant cells and blastopores are found. Blastopores are structures, such as cysts, that are approximately 30μm in diameter and are usually surrounded by daughter cells. Gomori-Grocott methenamine silver nitrate and periodic acid-Schiff staining are used to reveal the microorganism. Smears and culturing may also be used although they are inconvenient due to the slow rate of growth of the fungus [6].

Treatment involves anti-fungal medication (for example, amphotericin B, sulfadiazine, trimethoprim/sulfamethoxazole, ketoconazole, fluconazole, itraconazole) and supportive measures due to clinical complications associated with the involvement of different organs [7].

The duration of treatment depends on the severity of the disease and the type of medication used and is generally sufficient to enable long-term control of clinical symptoms and to prevent relapse. Patients are classified as cured when they meet the following clinical criteria: disappearance of the signals and symptoms, radiological stability (stabilization of the lung image pattern at three-month intervals) and serological stability (negative antibody titers or stabilization of values less than or equal to 1:2, observed in two samples collected at six-month intervals after the treatment period, recommended for itraconazole or trimethoprim/sulfamethoxazole).

After termination of treatment, as observed by the criteria for cure, patients should receive a follow-up examination once a year to obtain clinical and serological data. A positive titer increase or increase in the immunodiffusion reaction may precede clinical recurrence, which would warrant the reintroduction of anti-fungal therapy and further patient follow-up.

The absence of lesions in other organs and the localized symptoms (monoarthritis) exhibited by our patient in the present case were suggestive of several possible diagnoses which include septic arthritis, osteoarthritis, viral synovitis (toxic), hemophilia, giant-cell tumor, chondrolysis, tuberculosis and fungal etiology. Although the latter two potential diagnoses are rare, they are important and should not be overlooked.

The difficulty in diagnosing *P. brasiliensis* has been reported in the literature for non-disseminated cases [8-11]. In our study, this diagnosis was verified through biopsy and direct examination to identify the fungus, as recommended by various authors [12].

It should be emphasized that in patients presenting with pyoarthritis or osteomyelitis a diagnosis of paracoccidioidomycosis should be considered, especially for patients from rural areas in which the disease is endemic. Moreover, appropriate treatment should be provided as soon as possible in order to increase the likelihood of a more favorable prognosis following treatment.

## Conclusions

It is important to document the extreme rarity of such cases of pyoarthritis and osteomyelitis caused by local paracoccidioidomycosis. Cases involving seemingly immunocompetent pediatric patients who do not present lesions in other organs are poorly described in the literature, however paracoccidioidomycosis should be included in the differential diagnosis of osteoarticular infections, especially if the patient resides in a rural area where the disease is endemic.

## Consent

Written informed consent was obtained from the patient’s legal guardian for publication of this manuscript and any accompanying images. A copy of the written consent is available for review by the Editor-in-Chief of this journal.

## Competing interests

The authors declare that they have no competing interests.

## Authors’ contributions

MSM, MMM and RHR analyzed and interpreted the data from our patient regarding the infectious disease. LFF, EAF and SSM were the greatest contributors to the writing of the manuscript. All authors read and approved the final manuscript.
